# MULTIMORBIDITY AND ADL DISABILITY AMONG MEXICAN AMERICAN OLDER ADULTS: SEX DIFFERENCES

**DOI:** 10.1093/geroni/igae098.3455

**Published:** 2024-12-31

**Authors:** Eduardo Silveyra, Soham Al Snih

**Affiliations:** The University of Texas Medical Branch, Galveston, Texas, United States; University of Texas Medical Branch in Galveston, Galveston, Texas, United States

## Abstract

Medical advancements in the 20th century allowed the development of treatments for many diseases, increasing people’s average life expectancy. We examined sex differences in the relationship between multimorbidity and disability among Mexican American older adults ages ≥75 years who were non-disabled at baseline over 12-years of follow up. Participants (N=999) were from the Hispanic Established Population for the Epidemiologic Study of the Elderly (2004/2005-2016). Measures included socio-demographics, body mass index, pain, falls, depressive symptoms, cognitive and physical function, handgrip strength, multimorbidity and limitations in activities of daily living (≥ 1 ADL’s). Participants were divided into four groups according to the number of chronic conditions (CC): 0-1, 2, 3, and ≥ 4). Generalized Estimating Equation model were used to estimate the odds ratio and 95% Confidence Interval of ADL disability over time as a function of multimorbidity and number of chronic conditions controlling for all covariates. We found that female participants reporting 3 CC had greater odds of ADL disability (OR=1.60, 95% CI=1.08-2.38) over time than those with 0-1 CC after controlling for all covariates. Female and male participants reporting ≥4 CC had greater odds of ADL disability (OR=2.13, 95% CI=1.44-3.16 and OR=1.46, 95% CI=0.94-2.27, respectively) over time than those with 0-1 CC after controlling for all covariates. In conclusion, female participants were at increased risk for ADL disability when reporting ≥3 CC while the association was non-significant among males. Our findings indicate the need for evidence-based prevention and interventions targeting multimorbidity to prevent or delay ADL disability in this population.

